# De-Identification Technique with Facial Deformation in Head CT Images

**DOI:** 10.1007/s12021-023-09631-9

**Published:** 2023-05-25

**Authors:** Tatsuya Uchida, Taichi Kin, Toki Saito, Naoyuki Shono, Satoshi Kiyofuji, Tsukasa Koike, Katsuya Sato, Ryoko Niwa, Ikumi Takashima, Hiroshi Oyama, Nobuhito Saito

**Affiliations:** 1 Department of Neurosurgery, The University of Tokyo, 7-3-1 Hongo, Bunkyo-ku, Tokyo, 113-8655 Japan; 2 Department of Medical Information Engineering, Graduate School of Medicine, The University of Tokyo, 7-3-1 Hongo, Bunkyo-ku, Tokyo, 113-8655 Japan; 3 Data Science Office, Clinical Research Promotion Center, The University of Tokyo Hospital, 7-3-1 Hongo, Bunkyo-ku, Tokyo, 113-8655 Japan; 4 Department of Clinical Information Engineering, Graduate School of Medicine, The University of Tokyo, 7-3-1 Hongo, Bunkyo-ku, 113-8655 Tokyo, Japan

**Keywords:** Head CT images, Reconstructed face models, Personal information, De-identification, Deformation

## Abstract

Head CT, which includes the facial region, can visualize faces using 3D reconstruction, raising concern that individuals may be identified. We developed a new de-identification technique that distorts the faces of head CT images. Head CT images that were distorted were labeled as "original images" and the others as "reference images." Reconstructed face models of both were created, with 400 control points on the facial surfaces. All voxel positions in the original image were moved and deformed according to the deformation vectors required to move to corresponding control points on the reference image. Three face detection and identification programs were used to determine face detection rates and match confidence scores. Intracranial volume equivalence tests were performed before and after deformation, and correlation coefficients between intracranial pixel value histograms were calculated. Output accuracy of the deep learning model for intracranial segmentation was determined using Dice Similarity Coefficient before and after deformation. The face detection rate was 100%, and match confidence scores were < 90. Equivalence testing of the intracranial volume revealed statistical equivalence before and after deformation. The median correlation coefficient between intracranial pixel value histograms before and after deformation was 0.9965, indicating high similarity. Dice Similarity Coefficient values of original and deformed images were statistically equivalent. We developed a technique to de-identify head CT images while maintaining the accuracy of deep-learning models. The technique involves deforming images to prevent face identification, with minimal changes to the original information.

## Introduction

Data sharing is being widely promoted in medicine to advance research activities; however, there is increasing concern regarding the protection of personal information (El Emam et al., 2015; Gallagher, 2002; Huh, 2019; Poline et al., 2012; Raghupathi & Raghupathi, 2014; Scheinfeld, 2004; Vallance & Chalmers, 2013). Medical images such as head CT can be used to reproduce human facial features using 3D reconstruction (Bischoff-Grethe et al., 2007; Budin et al., 2008; Chen et al., 2014; Matlock et al., 2012; Mazura et al., 2012; Milchenko & Marcus, 2013; Prior et al., 2009; Theyers et al., 2021). The Health Insurance Portability and Accountability Act 2006 summit in the United States highlighted that 3D-reconstructed facial information could be personally identifiable (Steinberg, 2006). Tremendous changes in communication methods between medical professionals make it necessary to consider legal, social, and ethical issues regarding protecting personal information (Lamas et al., 2015; Lamas et al., 2016; Voßhoff et al., 2015).

Various studies indicate the risk of 3D facial reconstruction of head medical images in identifying patient faces (Chen et al., 2014; Mazura et al., 2012; Parks & Monson, 2017; Prior et al., 2009; Schwarz et al., 2019; Schwarz et al., 2021).

Typical existing de-identification techniques include deleting facial voxels (QuickShear, Defacing) (Bischoff-Grethe et al., 2007; Matlock et al., 2012), local deformation (Facial Deformation) (Budin et al., 2008), blurring (Face Masking) (Milchenko & Marcus, 2013), and face replacement (Reface) (Schwarz et al., 2021).

QuickShear and Defacing aim to prevent facial identification by deleting voxels in the face region and eliminating facial information. Loss of anatomical structures on the surface and inside the face results in considerable alteration of information and could impede data utilization (de Sitter et al., 2020). Removal of the entire face makes detecting faces from the reconstructed face models difficult. Images in which faces cannot be detected from the reconstructed face models have been shown to reduce the accuracy of medical image alignment, such as affine transforms. It has various effects on medical image processing which may be problematic for use in surgical simulation and research. Facial Deformation only deforms characteristic facial regions and preserves the internal anatomy. Achieving sufficient de-identification using this technique requires such great deformations that the face shape cannot be preserved, making face detection from reconstructed models difficult. Face Masking applies smoothing of the face surface to prevent face identification; preventing face identification in this way requires such strong blurring that the anatomical structure of the face surface cannot be preserved, making face detection difficult. Reface performs face removal and then replaces the face with another face. In this technique, the change in information is so great that boundary areas between the original and additional images are created, tissue continuity is not maintained, and the integrity of the internal anatomical structures is greatly disrupted.

A report revealed that images processed with QuickShear, Defacing, and Face Masking for segmenting brain tissue and tumors achieved reduced output accuracy in deep learning models (de Sitter et al., 2020).

We aimed to develop a new de-identification technique whereby post-processing reconstructed face models are face-detectable, pre- and post-processing reconstructed face models are not considered for the same person, pre-and post-processing information changes are small, and post-processing images are suitable for deep learning.

## Materials and Methods

### Acquisition of CT Images

**Fig. 1:**
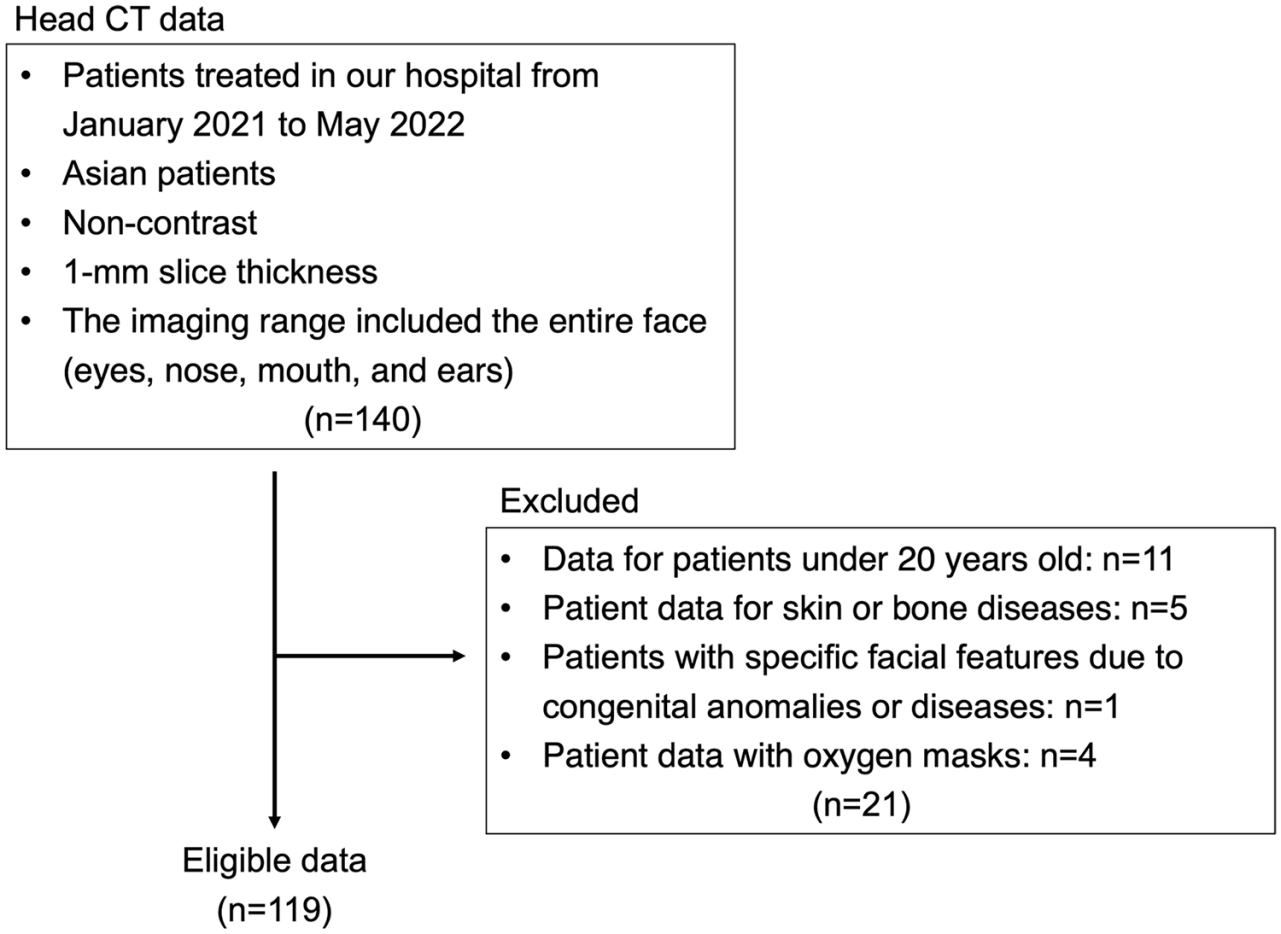
Flowchart for selection of eligible data in this development studies. A total of 140 non-contrast head CT images were obtained, and 119 images were included

Non-contrast head CT images of 140 Japanese patients and volunteers admitted to our hospital between January 2021 and May 2022 were obtained. CT was performed using a 320-row multi-slice CT scanner (Aquilion ONE; Toshiba Medical Systems, Tokyo, Japan), using the following parameters: collimation, 0.5 mm; tube voltage, 120 kV; tube current, 200 mA; rotation time, 0.6 s; reconstruction section width, 0.5 mm; reconstruction interval, 0.5 mm; and voxel size, 0.43 × 0.43 × 1.00 mm. The imaging range included the entire face ( eyes, nose, mouth, and ears). Images of patients < 20 years old (11 cases), patients with prominent skin or bone lesions (five), patients with specific facial features due to congenital anomalies or disease (one), and patients wearing oxygen masks (four) were excluded; 119 head CT images were included (Fig. 1). All procedures involving human participants were in accordance with the 1964 Helsinki declaration and its later amendments. The Institutional Review Board of our hospital approved the study protocol (approval number: #2021107NI). Written informed consent was obtained from all patients before participation.

### Methods for Creating Reconstructed Face Models

Head CT images were input into the image processing software Amira 3D^®^ (version 2021.1; Thermo Fisher Scientific, Waltham, MA, USA) (Thermo Fisher Scientific, 2022) using Digital Imaging and Communications in Medicine format to create reconstructed face models. The images were processed using the marching cubes algorithm with a simple thresholding method and visualized using the surface rendering method. Directional lighting was used, the object color scheme was gray, and the threshold was set to -200. Other than the head, the human body and head fixtures were deleted. Snapshots (.png) in the forward-facing position (arbitrarily set by the creator, a board-certified neurosurgeon) were used as visual information.

### Methods of Deformation Processing

**Fig. 2:**
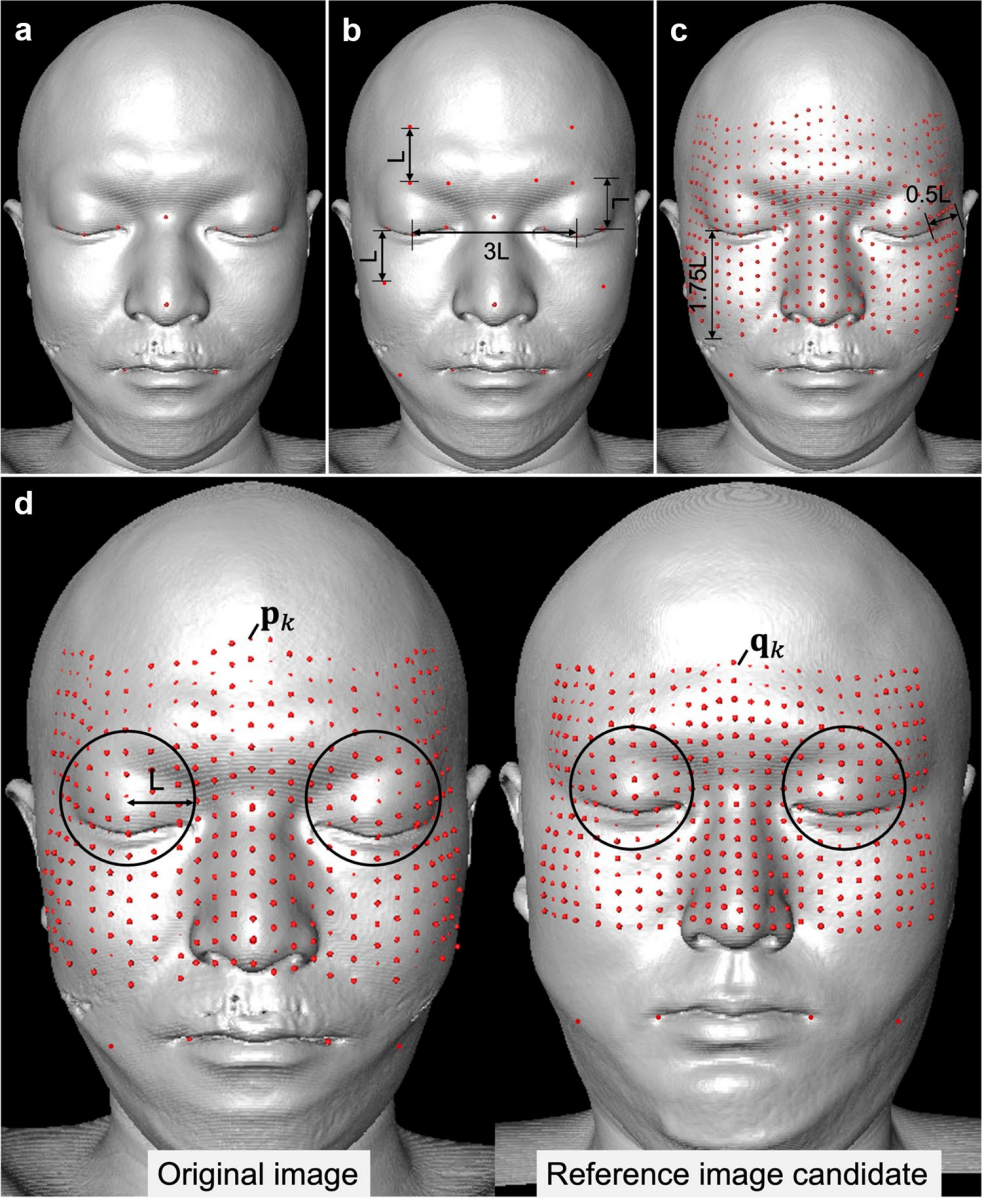
Procedure for setting control points and selecting a reference image. **a** Ten points were set as standard control points: inner canthus, outer canthus, midpoints, nasal root, nasal tip, and both corners of the mouth. **b** Four control points were added for the eyebrows, two for the forehead, two for both cheeks, and two for the chin, for 20 points. **c** Control points were added in a grid-like pattern to the area bounded by the set control points, and the area was expanded in the lateral direction of the outer canthus. The additional control points were limited to the area above the mouth for 400 points. **d** The area around the eyes is defined as the range within a sphere of radius L centered at the midpoint between the eyes and eyebrows. The image with the largest summed distance between the corresponding control points around the eye between the two images was used as the reference image. L: baseline distance (1/3 of the distance between the midpoints of both eyes), $p$: control point of the original image, $q$: control point of the reference image candidate, $k$: index of the control point

Standard control points (10 points) were set on the feature parts of the face (Fig. 2a).

- Eye: six points in total on the internal, midpoints, and external areas
- Nose: two points on the root and tip
- Mouth: two points on both corners of the mouth

One-third of the distance between the midpoints of both eyes was used as the baseline distance (L), and the following 10 control points were added at locations along the geodesic line above and below the set control points (Fig. 2b).

- Eyebrow: four points on the midpoint (above the midpoint of the eyes) and the medial side (above the inner canthi)

-Forehead: two points on the midpoint (above the midpoint of the eyebrows)

- Cheek: two points on the lower part of the outer canthus
- Chin: two points on the corner line of the mouth below the outer canthus

In a grid-like pattern, additional control points were added to the area bounded by the 20 set control points. The range of control points to be added was extended by 0.5 L in the lateral direction of the outer canthi. The added control points were limited to the area above the mouth to minimize the influence of intraoral metal artifacts. Finally, 400 control points were set (Fig. 2c). All control points were set manually using Amira 3D^®^.

The 118 images other than the original image to be transformed were considered as the reference image candidate group, and the reference image was selected from among these images. The image with the greatest difference in shape around the eyes compared to the original image was selected as the reference image to deform the face and resemble a different face. Details of the reference image selection method are provided below.

Based on the assumption that the control points were set for the original image and all the reference images, registration between the original image and the reference image candidates was first performed. Scale alignment between the original image and reference image candidates was performed as a preprocessing step for registration. The scale of the reference image candidates was enlarged or reduced such that the distance between the midpoints of both eyes between the original image and the reference image candidates was equal.

After scale alignment, the corresponding points (midpoints of the eyes, eyebrows, and forehead, six points in total for each image) were set for the original image and the reference image candidates, and registration was performed using the least-squares method (Abdi, 2007; Jiang, 1998), which minimizes the sum of the distances between the corresponding points. Because rigid body registration with six degrees of freedom (three translation degrees of freedom and three rotation degrees) was used, no interpolation was performed for the surface points between the control points.

Next, control points around the eyes were selected. The area around the eyes was defined as the range contained within a sphere of radius L (1/3 of the distance between the midpoints of both eyes) centered at the midpoint between the eyes and the eyebrows (Fig. 2d). Control points around the eyes were selected for each of the original and reference image candidates. The sum of the distances (*D*) between the corresponding control points in the two images was calculated.

1 $$D={\sum }_{k}^{n}{\left|{\mathrm{q}}_{k}-{\mathrm{p}}_{k}\right|}^{2}$$

*p* is the control point of the original image, *q* the control point of the reference image candidate, *k* the control point index, and *n* the number of control points used in the evaluation. The image with the largest *D* is the reference image *R*, which is given by the following formula:

2 $$R=arg \underset{i}{max }D\left(i\right)$$

*i* is the index of the reference image candidate and *D(i)* is the sum of the distances between the control points when the reference image candidate *i* is used as the reference image.

Based on the assumption that registration of the original and reference images was complete, voxels in the original image were moved according to the following procedure.

First, to deform the control points in the original image to the corresponding control points in the reference image, the deformation vector *d* of each control point is calculated using the following formula:

3 $${d}_{k}={q}_{k}-{p}_{k}$$

*p* is the control point in the original image, *q* is the control point in the reference image *R*, *d* is the deformation vector of the control point, and *k* is the index of the control point.

Next, the deformation vector *C*_*j*_ at the voxel coordinate *I*_*j*_ was calculated by computing a weighted addition of the deformation vectors of the surrounding control points using the following formula:

4 $${C}_{j}=\frac{{\sum }_{k}^{n}\left(G\left(\left|{{p}_{k}-I}_{j}\right|,{\sigma }_{2}\right){w\left(\left|{{p}_{k}-I}_{j}\right|,{\sigma }_{1}\right)d}_{k}\right)}{{\sum }_{k}^{n}G\left(\left|{{p}_{k}-I}_{j}\right|,{\sigma }_{2}\right)}$$

5 $$G\left(x,\sigma \right)=\frac{1}{\sqrt{2\pi }\sigma }exp\left\{-\frac{{x}^{2}}{2{\sigma }^{2}}\right\}$$

6 $$w\left(x,\sigma \right)=\frac{G\left(x,\sigma \right)}{G\left(0,\sigma \right)}$$

*C* is the deformation vector of each voxel in the original image, *n* is the number of control points, *I* is the voxel coordinates in the original image, *j* is the index of voxels in the original image, *σ*_*1*_ and *σ*_*2*_ are the standard deviations, *G* is the normal distribution, and *w* is the normal distribution divided by the center value. This formula shows that the deformation vectors of nearby control points affect *C* more when performing a weighted addition.

**Fig. 3:**
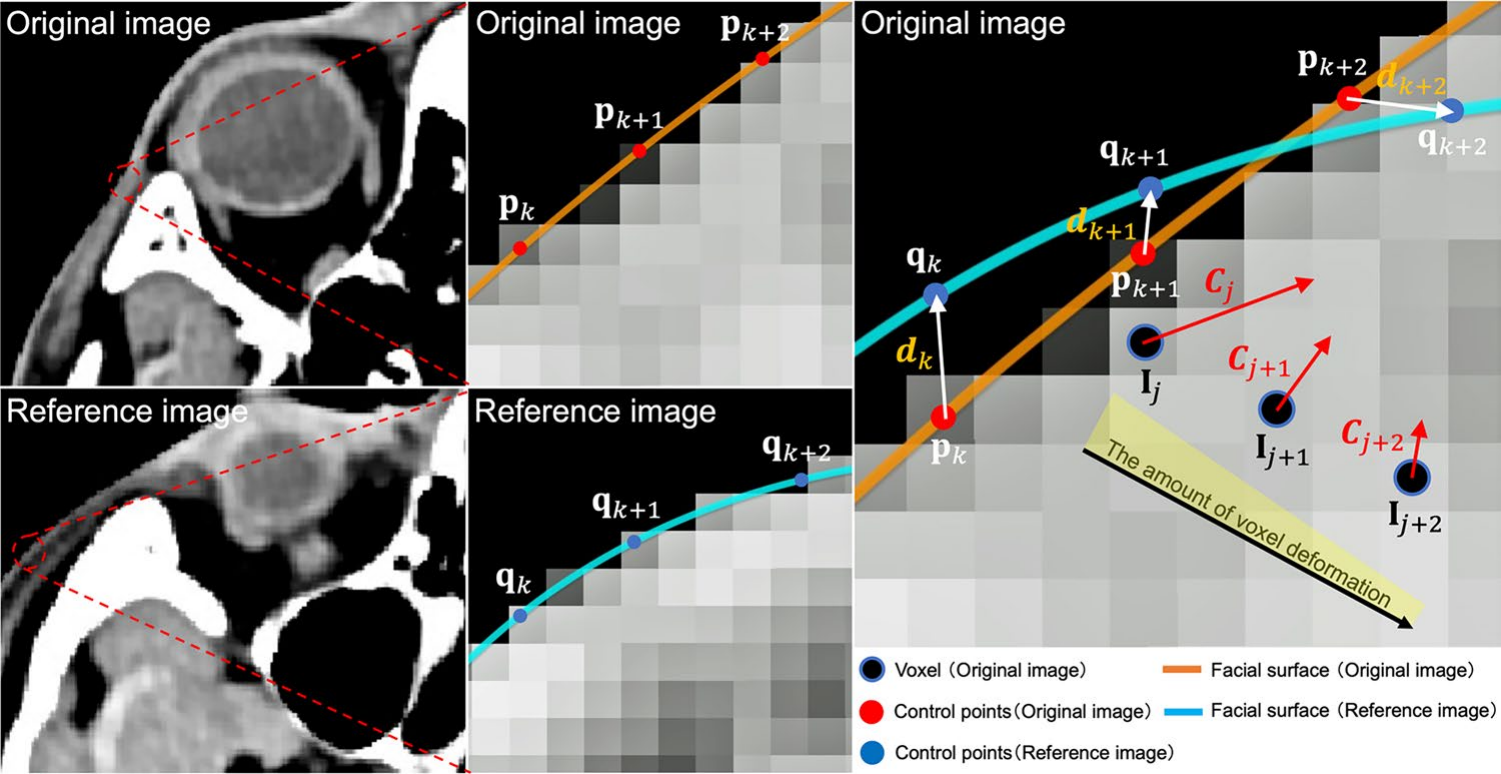
Movement of control points and voxels. The voxels in the original image are moved based on the deformation vector calculated for each control point. The closer the voxel is to the control points, the greater the deformation; the further away the voxel is from the control points, the more it decays. In addition, when adding voxel deformations, the closer the control points, the stronger the effect. $C$: deformation vector of each voxel in the original image; $d$: deformation vector of the control point; $I$: voxel coordinates in the original image; $j$: index of voxels in the original image; $k$: index of the control point; $p$: control point of the original image; $q$: control point of the reference image

The deformation vectors of all voxels in the original image were calculated, and the deformed image was obtained by moving the voxel positions according to the deformation vectors (Fig. 3). The amount of voxel deformation was attenuated with the distance from the control points. 1σ was set to 7.5 mm, and the effect on voxels at distances greater than 3σ was ignored.

### Evaluation of Accuracy

1. Face detection test

Face detection tests were conducted using face detection programs provided by three software packages: Face API (Microsoft Corp., Redmond, WA, USA) (Azure Microsoft, 2022), Rekognition (Amazon.com, Inc, Seattle, WA, USA) (Amazon, 2022), and NeoFace KAOATO (NEC Corp., Tokyo, Japan) (NEC solution innovator, 2022). The face detection rate (percentage of faces detected from 119 cases) was calculated.

2. Face identification test

Face identification tests were conducted using the face identification programs provided by the same software packages. Face identification programs detect the feature points of each face and output the match confidence score, which indicates the confidence level that the two faces are the same person, based on the coincidence of these feature points.

**Fig. 4:**
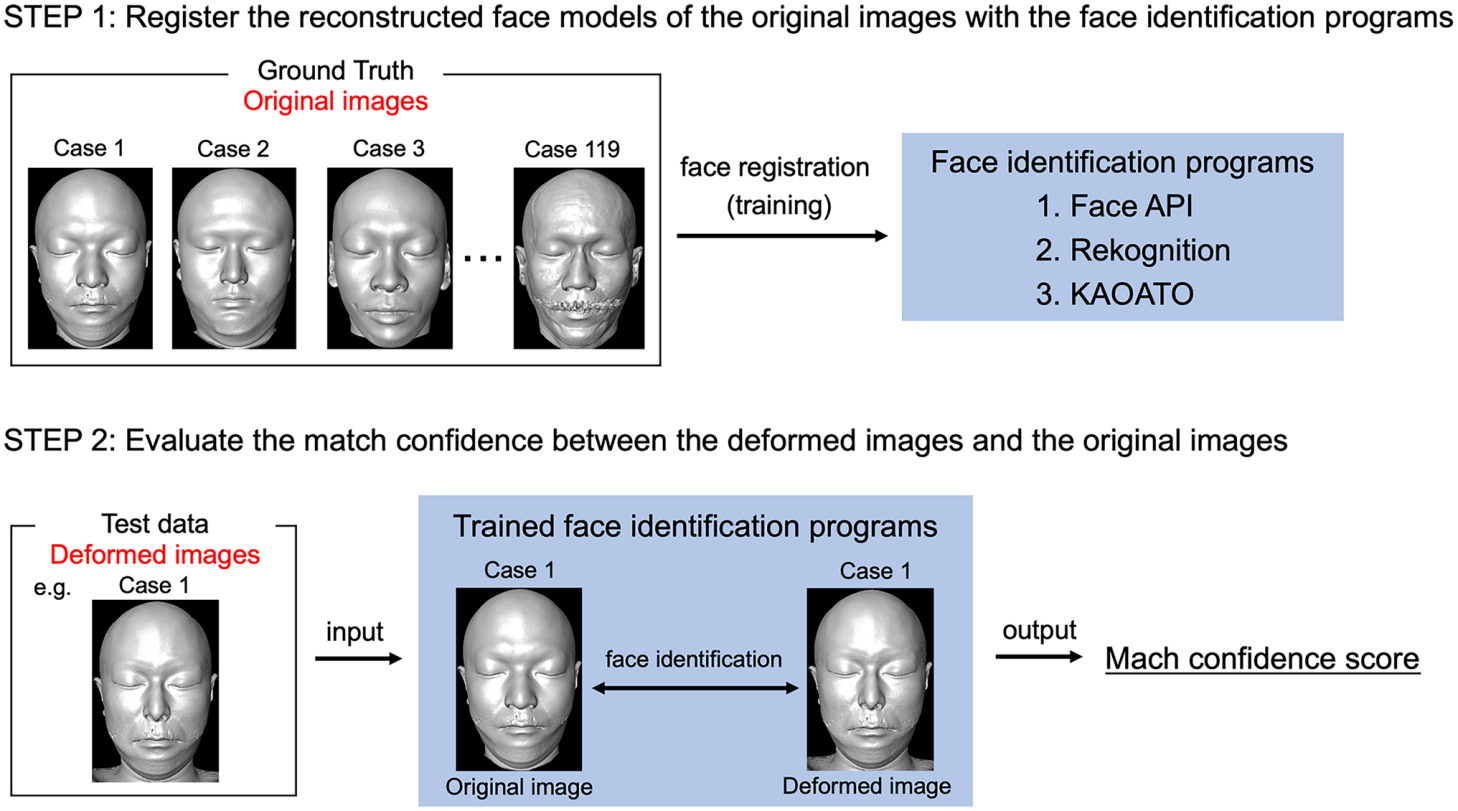
Face identification test flow. The reconstructed face models of the original images were registered with the three face identification programs as Ground Truth (STEP 1). The reconstructed face model of the deformed image was input into the programs as test data. The match confidence score between the original and deformed images of the same person was obtained (STEP 2)

In the reconstructed face models of the original images, seven snapshots (.png) for each person at different angles were taken: straight ahead, 10° and 20° left and right, and 10° up and down (119 cases, 833 snapshots). They were registered with the program Ground Truth. A snapshot of the reconstructed face model (straight ahead) of the deformed image was input into the programs as test data. Subsequently, the match confidence score between the original and deformed images of the same person was obtained. Match confidence scores are output in the range 0–100 (0: absolute disagreement, 100: absolute match) (Face API outputs the match confidence scores in the range of 0–1, so the output results are multiplied by 100 to standardize the range). In all 119 cases, the match confidence scores were verified (Fig. 4).

3. Verification of information changes

Intracranial volume was measured using image processing software using Amira 3D^®^. A seed was set in the thalamus, and intracranial segmentation was performed with thresholds ranging from -50 to 120. Unnecessary soft tissue and the spinal cord below the inferior end of the frontal lobe were removed. The volume of the segmentation area was measured. In all cases, intracranial volumes of the original and deformed images were measured and compared.

The similarity between the intracranial pixel-value histograms of the original and deformed images was verified. Intracranial regions in the two images were segmented using the method described above, and only the segmented intracranial regions were extracted from the volume data. The correlation coefficient (Guilford, 1956) between the two images was calculated using the normalized correlation to evaluate the similarity of the intracranial pixel value histograms in the images before and after deformation. The image processing software Amira 3D^®^ was used for the entire process and calculation of the correlation coefficients. The correlation coefficient (**r**) is given by the following formula:

7 $$r= \frac{{\sum }_{i=1}^{n}\left(\overline{M}-{M}_{i}\right)\left(\overline{I}-{I}_{i}\right)}{\sqrt{{\sum }_{i=1}^{n}{\left(\overline{M}-{M}_{i}\right)}^{2}}\sqrt{{\sum }_{i=1}^{n}{\left(\overline{I}-{I}_{i}\right)}^{2}}}$$

4. Verification of utilization

A deep learning model for intracranial segmentation was created using the Dragonfly^®^ software (version 2021.3; Object Research Systems, Montreal, Canada) (Research Systems Object, 2022), which can create deep learning models for medical image segmentation. Of the 119 eligible images, 100 were used as training data, and 19 were used as test data.

**Fig. 5:**
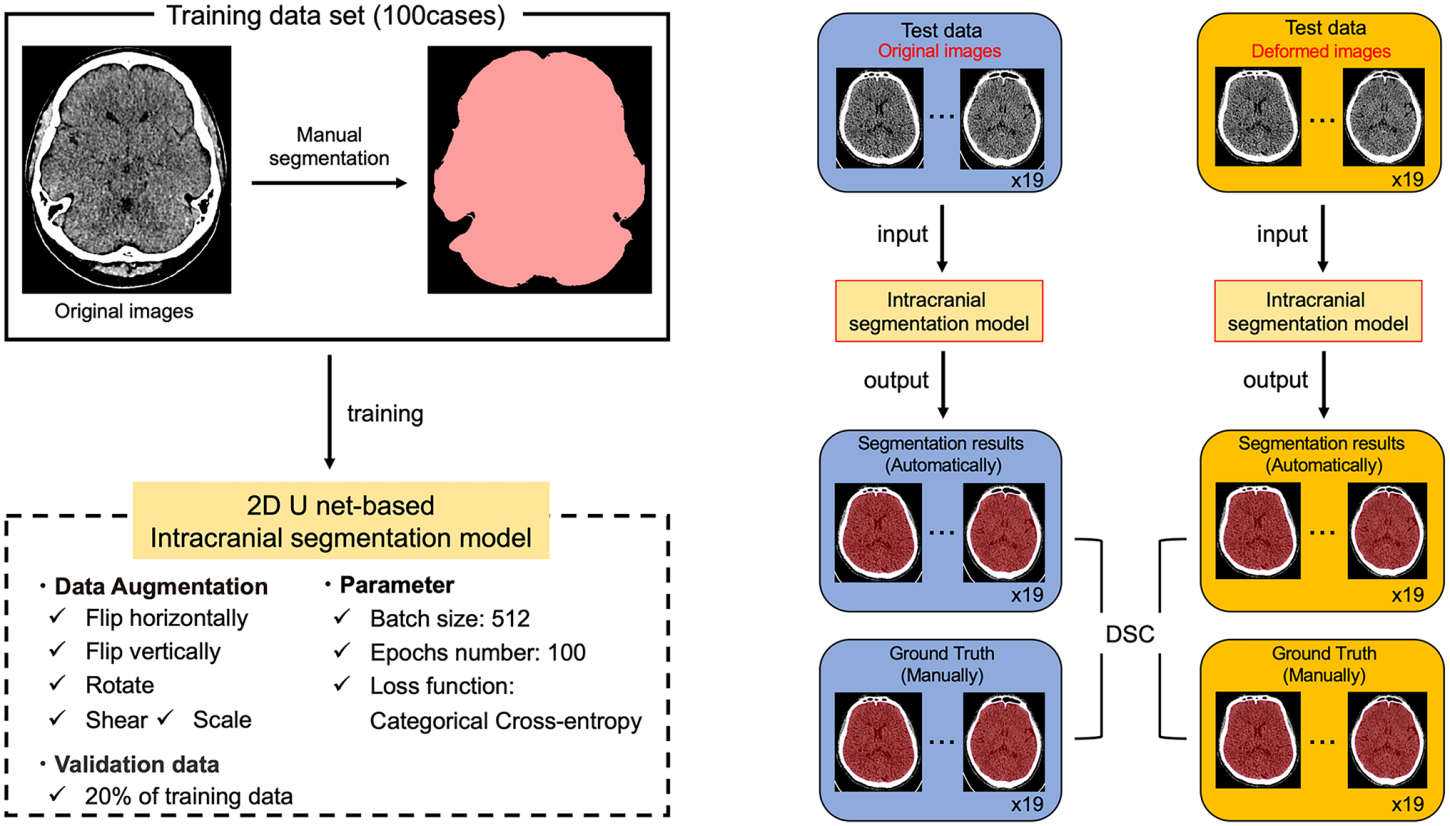
Output accuracy of deep learning models for intracranial segmentation. Left: The 100 cases training dataset were trained on 2D Unet to create a deep learning model. Right: Nineteen cases, each with a corresponding original and the deformed image, were used as test data. The DSC was calculated by inputting the test data into a deep-learning model for intracranial segmentation. DSC for the original images and deformed images input were calculated and compared, respectively. DSC: Dice Similarity Coefficient

The intracranial region was segmented in the same way as when the intracranial volume was measured and outputted as binary data (1: intracranial, 0: other regions), and the Ground Truth was created. The original images and Ground Truth were used as the training dataset (100 cases) and were trained on the 2D U-net (Ronneberger et al., 2015; Presotto et al., 2022). Data augmentation was performed by flipping horizontally and vertically, rotating, shearing, and scaling. The parameters were a batch size of 512, an epoch number of 100, and categorical cross-entropy as the loss function. For the validation data, 20% of the training data was used (Fig. 5).

The Dice Similarity Coefficient (DSC) (Dice, 1945) was used to evaluate the output accuracy of the deep learning model. In the original and deformed images, each of the 19 images not used for training was input into the deep learning model as test data. DSC was calculated and compared between the original and deformed image inputs (Fig. 5).

## Statistical Analysis

Equivalence tests (Dunnett & Gent, 1977) were performed to verify whether the intracranial volumes of the original and deformed images were equivalent. The equivalence margin was set at 1% of the mean intracranial volume of the original image, with a significance level of 5%. Brand–Altman analysis (Bland & Altman, 1986) was performed to verify the equivalence of the DSCs for the original and deformed images. Statistical analyses were performed using the JMP Pro^Ⓡ^ 16 (SAS Institute Inc., Cary, NC, USA).

## Results

Head CT images were acquired from 119 participants. The study participants were 48 men and 71 women (mean age: 55 ± 18 [range: 21–92] years). The clinical characteristics of participants are presented in Table 1. The clinical diagnoses were cerebrovascular diseases in 34 (29%), brain tumors in 54 (45%), functional diseases in 22 (18%), and others in 9 (8%) cases.

**Table 1:** Characteristics of Participants

**No. of participants**	119
Age (years)*	55 ± 18 [21–92]
**Sex**	
Female	71 (60)
Male	48 (40)
**Diagnosis**	
Cerebrovascular disease	34 (29)
Brain tumor	54 (45)
Functional disorder	22 (18)
Others	9 (8)

Except where indicated, data are numbers of patients, with percentages in parentheses

*Age is expressed in years as the mean ± standard deviation, with the range in brackets

### Deformation Processing

**Fig. 6:**
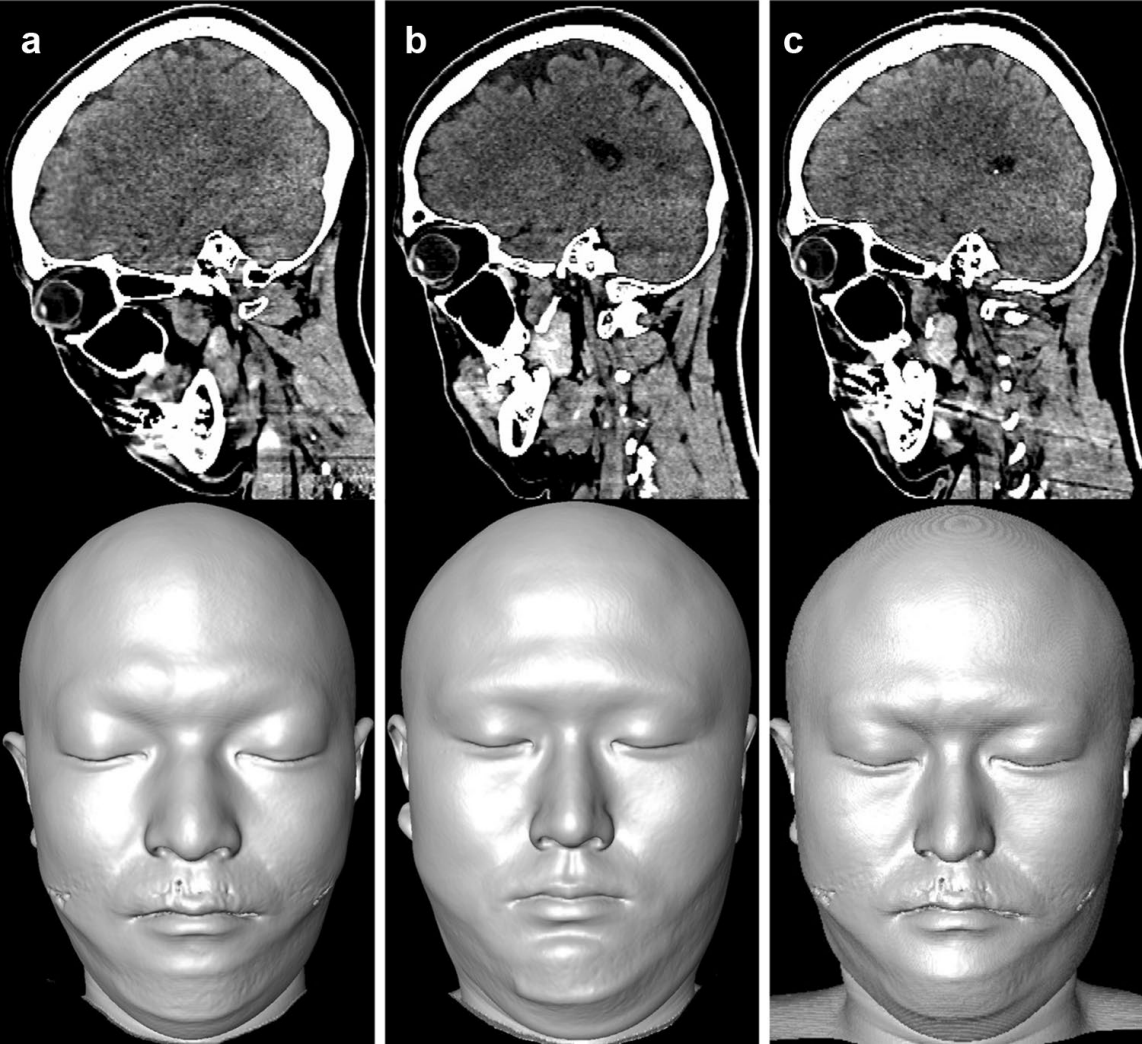
Deformation processing for the original image. Top: CT volume data, Bottom: reconstructed face models from above images. **a** original image, **b** reference image, and **c** deformed image. In all cases, the control points could be set, the reference images were selected, and the deformed images were created

In all 119 cases, facial reconstruction models were created, and control points were set. Reference images could be selected in all cases, and deformation processing was performed on all original images using the selected reference images. The original, reference, and deformed images of one case are shown in Fig. 6.

### Evaluation of Accuracy

The face detection rate was 100% in all face detection programs for both reconstructed face models of the original images and those of deformed images.

**Fig. 7:**
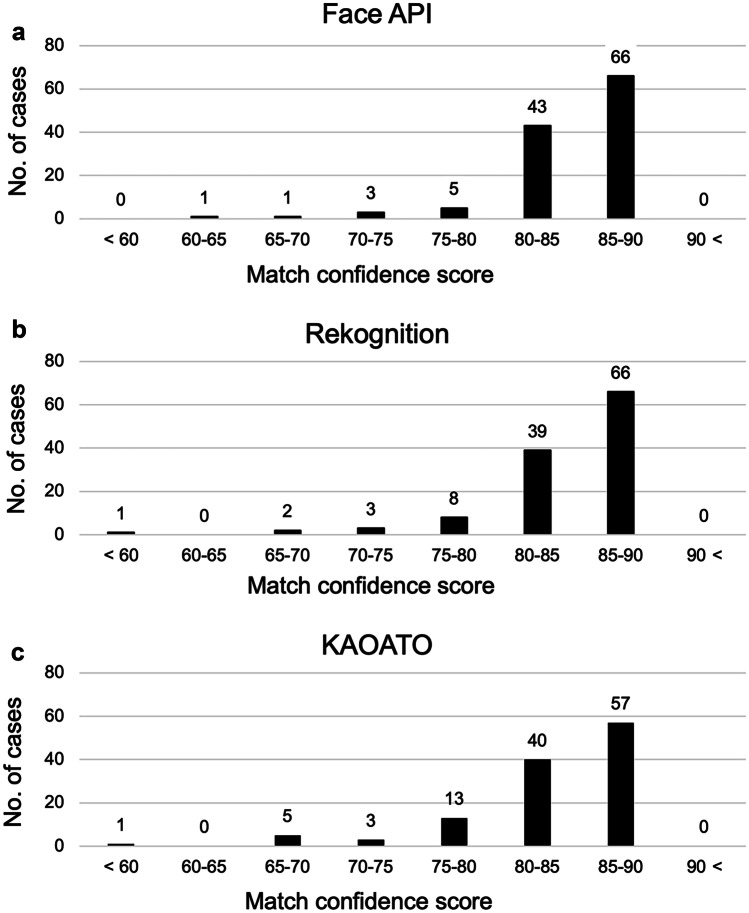
Match confidence scores distribution for each face identification program. Distribution of match confidence scores in **a** Face API, **b** Rekognition, and **c** NeoFace KAOATO. All face identification programs had match confidence scores below 90 in all cases, and there were no significant differences among the three programs

The distribution of the match confidence scores is shown in Fig. 7. The median match confidence scores were 86.77 (interquartile range: 84.16–87.93) for Face API (Fig. 7a), 85.36 (interquartile range: 83.29–87.70) for Rekognition (Fig. 7b), and 84.59 (interquartile range: 81.07–88.04) for NeoFace KAOATO (Fig. 7c). In all cases, the match confidence scores were less than 90 for all face identification programs, and the distribution of match confidence scores did not differ among the three programs.

The mean ± standard deviation of the intracranial volume in the original images was 1412 ± 148 (×10^3^ mm^3^), and that in the deformed images was 1410 ± 152 (×10^3^ mm^3^).

**Fig. 8:**
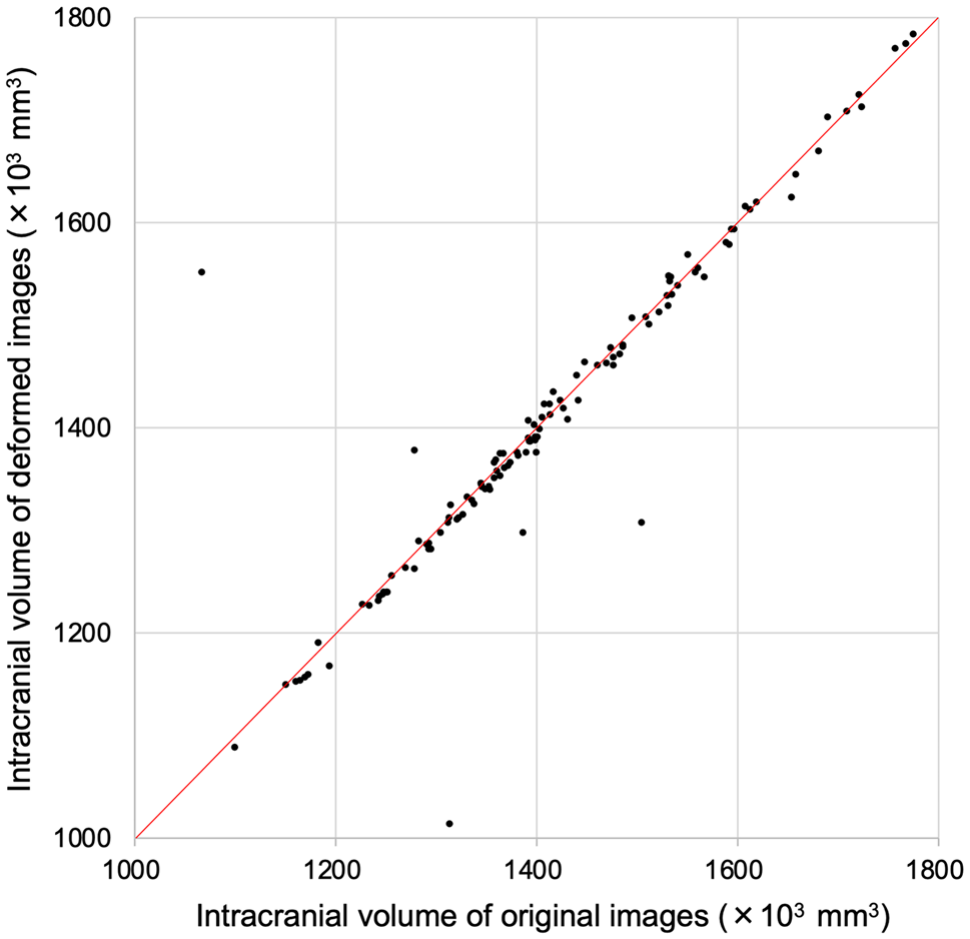
Relationship between intracranial volume in original and deformed images. The intracranial volume changes due to deformation are shown in the scatter plot. The red line represents the identity line that shows a perfect match

The relationship between the intracranial volumes of the original and deformed images for each case is shown in Fig. 8. An equivalence test was performed using 1% of the mean intracranial volume in the original images, 14 × 10^3^ mm^3^ as the equivalence margin. Statistical analysis shows that the difference between the means is "above the lower limit (-14 × 10^3^ mm^3^) (P = .0148)" and " below the upper limit (14 × 10^3^ mm^3^) (P = .0012)," indicating that the intracranial volume means between the original images and the deformed images were statistically equivalent; in other words, the two mean volumes were within a certain range. The % variance in intracranial volume difference was 105.5%, a small increment in the deformed images, but not an important difference.

The median correlation coefficient was 0.9965 (interquartile range: 0.9951–0.9974), and the intracranial pixel value histograms between the two images showed high similarity in all cases (Table 2).

**Table 2:** Correlation coefficient of intracranial pixel value histograms

**Correlation coefficient**	**No. of cases**
> 0.998	14 (12)
0.996–0.998	58 (49)
0.994–0.996	31 (26)
0.992–0.994	7 (6)
< 0.992	9 (7)

The intracranial pixel value histograms between the original and deformed images were higher than 0.990 in all cases, indicating a high degree of similarity. Data are numbers of cases, with percentages in parentheses

**Fig. 9:**
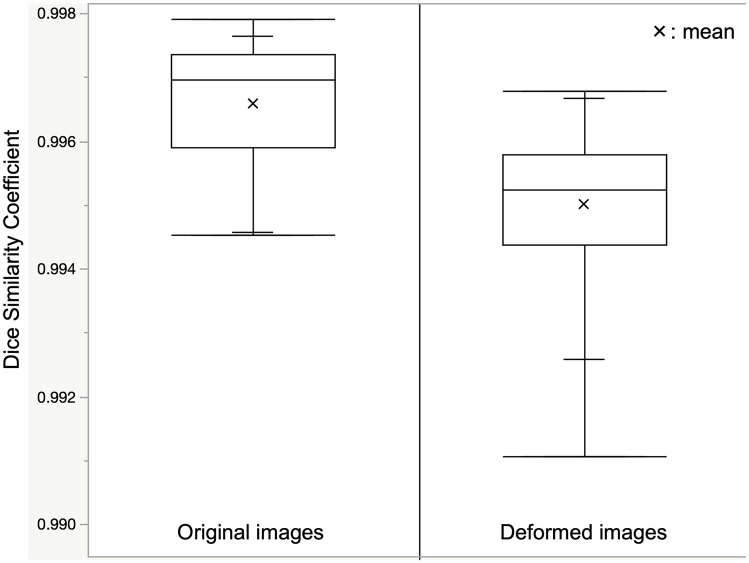
Distribution of the Dice Similarity Coefficient. Distribution of the Dice Similarity Coefficient (DSC) when the original and deformed images are input to the deep learning model for intracranial segmentation. The Brand–Altman analysis showed that the mean difference in DSCs in all cases was within the acceptable error margin. The DSCs of the original and deformed images could be interpreted as being close to equivalent

The original and deformed images were input to the deep learning model for intracranial segmentation, and the DSC for the original and deformed images was calculated for all 19 cases. The DSC distribution is shown in Fig. 9. The median DSC was 0.9967 (interquartile range: 0.9962–0.9973) for the original images and 0.9952 (interquartile range: 0.9945–0.9958) for the deformed images, both with a high DSC. A Brand-Altman analysis was performed to compare the DSCs of the original and deformed images. The mean differences in the DSCs between the two images were within an acceptable error margin (95% confidence interval). The DSCs of the original and deformed images were nearly statistically equivalent.

## Discussion

### Study Results Summary

Using control points, a new de-identification technique was developed in head CT images to deform original images to resemble reference images. The reconstructed face models of the deformed images were face-detectable and provided sufficient facial changes from the original images. The intracranial volume and pixel value histograms were equivalent before and after deformation. The output accuracy of the deep learning model for intracranial segmentation was equivalent to the original and deformed images.

Importantly, all the deformed reconstructed face models could likely detect faces because using another person's images as a reference guaranteed that the destination was a human face, even if the control points moved significantly.

According to guidelines (European Data Protection Board, 2022) provided by the European Data Protection Board, the threshold for match confidence scores in critical security situations involving personal information, such as police and banks, is recommended to be ≥ 90 to consider it as the same person. However, no appropriate thresholds for face identification tests between reconstructed face models have been reported. Here, a match confidence score < 90, generally judged to indicate a different person, was accepted as a change in the face. The match confidence scores were < 90 for all face identification programs, possibly because the facial features of the original images were sufficiently altered to the facial features of the reference images, and the deformations were performed to achieve a closer resemblance to the faces of the reference images.

Because the accuracy of face identification by human visual assessment has been reported to be significantly less than that of face identification programs (Chen et al., 2014; Prior et al., 2009), a human visual assessment was not performed.

Intracranial volume has been reported to decrease by approximately 10% between 40 and 75 years old (Fillmore et al., 2015); therefore, the effect of a volume change < 1% in this study was considered sufficiently small. The correlation coefficients of the intracranial pixel value histograms between the original and deformed images were higher than 0.9, indicating a robust correlation. One reason for the small changes in intracranial information before and after deformation may be that the deformation was attenuated with distance from the face surface, so the deformation effect was smaller in the interior. The reasons for the equivalence of the DSCs could be the absence of high-impact processing, such as deletion and blurring, the suppression of internal deformations, and the absence of unnatural boundary areas.

### Strengths and Novelties of this Technique

The problems with existing techniques are that face detection is no longer possible, substantial information changes occur during processing, and the output accuracy of deep learning models related to medical image segmentation is reduced. No technique has overcome all these problems. The de-identification technique proposed in this study has the potential to be more useful regarding face detection, information changes, and maintaining the accuracy of deep learning models. The main advantage of our technique over existing techniques is that no unnatural boundaries are generated, consistency is maintained, and the deformed image is indistinguishable from the original one when compared.

If de-identification techniques involve excessive processing, too much information is lost, and the processed images are impractical to use as research material. Conversely, if the degree of processing is too small, there is a greater concern that the face may be identified. There has always been a trade-off between information preservation and face identification prevention. The three main novelties of this technique are that control points were set for the deformation of the face surface, a reference image was used to move the control points, and the degree of deformation was attenuated according to the distance from the control points. Using the reference images, the shape of the face was preserved regardless of how far the control points moved. Because the control point was located only on the face surface, the deformation was attenuated according to the distance from the control points, thereby minimizing changes to intracranial information.

### Limitations and Future Work

This study had several limitations. Only CT images were used, but in the future, the technique must also be applied to MRI to confirm its accuracy.

In medical images, there is no established method to evaluate the success or failure of de-identification of medical images, and the degree of deformation that can be said to be "a different face" in the facial reconstruction model is unknown. We reported only the technical details of de-identification, and it cannot be strictly asserted that the results of this study make it possible to legally anonymize the data. The concept of "ELSI" is considered an essential nontechnical issue when developing medical technology and sharing medical data (Fisher, 2005). ELSI indicates "Ethical, Legal, and Social Issues" and advocates the need to discuss ethical issues and their impact on individuals and society when new methods and technologies not previously available are not addressed by current laws. The proposed de-identification technique could contribute to the social and ethical treatment of personal information, even if legal interpretation is challenging.

## Conclusion

A new de-identification technique was developed in head CT images to deform original images to reference images using control points. In deformed images, the reconstructed face models exhibited detectable and sufficient facial changes from the original in all cases; intracranial volume and intracranial pixel value histograms were equivalent before and after deformation. The output accuracy of the deep learning model for intracranial segmentation was equivalent to the original and deformed images.

## Data Availability

Data generated or analyzed during the study are available from the corresponding author by request.

## Declarations

### Information Sharing Statement

Data are not publicly available. Amira 3D® is available at https://www.thermofisher.com/jp/en/home/electron-microscopy/products/software-em-3d-vis/amira-software/cell-biology.html. Face API is available at https://azure.microsoft.com/en-gb/products/cognitive-services/face. Rekognition is available at https://aws.amazon.com/rekognition/?nc1=h_ls. NeoFace KAOATO is available at https://www.nec-solutioninnovators.co.jp/sl/kaoato/index.html. Dragonfly is available at http://www.theobjects.com/dragonfly/.
